# The dinitrobenzamide mustard prodrugs, PR-104A and SN27686, for use in a novel MNDEPT cancer prodrug therapy approach

**DOI:** 10.1042/BSR20230627

**Published:** 2023-04-25

**Authors:** Patrick Ball, Emma Thompson, Simon Anderson, Vanessa Gwenin, Amir Ashoorzadeh, Jeff Smaill, Chris Gwenin

**Affiliations:** 1 School of Natural Sciences, Bangor University, Bangor, Gwynedd, Wales LL57 2UW, U.K.; 2 Auckland Cancer Society Research Centre, School of Medical Sciences, The University of Auckland, Private Bag 92019, Auckland 1142, New Zealand; 3 Maurice Wilkins Centre for Molecular Biodiscovery, The University of Auckland, Private Bag 92019, Auckland 1142, New Zealand; 4 Division of Health Sciences, Abertay University, Bell Street, Dundee, DD1 1HG, U.K.

**Keywords:** cancer, DEPT, nitroreductase, prodrug, therapy

## Abstract

Directed enzyme prodrug therapy is a highly promising anti-cancer strategy. However, the current technology is limited by inefficient prodrug activation and the dose-limiting toxicity associated with the prodrugs being tested; to overcome these limitations, the dinitrobenzamide mustard prodrugs, PR-104A and SN27686, have been developed. The present study will assess both of these prodrugs for their potential uses in a novel magnetic-nanoparticle directed enzyme prodrug therapy strategy by determining their kinetic parameters, assessing the products formed during enzymatic reduction using HPLC and finally their ability to cause cell death in the ovarian cancer cell line, SK-OV-3. It was shown for the first time that the dinitrobenzamide mustard prodrugs are able to be reduced by the genetically modified nitroreductases, NfnB-cys and YfkO-cys, and that these enzyme/prodrug combinations can induce a significant cell death in the SK-OV-3 cell line, highlighting the potential for both enzyme/prodrug combinations for use in magnetic-nanoparticle directed enzyme prodrug therapy.

## Introduction

Cancer is one of the leading causes of global deaths each year and is typically treated using one of three methods: surgery, radiotherapy or chemotherapy. While all three methods have been refined over the years, the lack of selectivity between healthy and cancerous cells continues to be a major limitation, particularly when it comes to chemotherapy strategies [1,2]. Efforts to overcome this limitation have led to the development of new therapeutic approaches that direct the treatment to the cancer site, using a targeting system, such as by using a directed enzyme prodrug therapy (DEPT) approach [3,4]. DEPT treatments involve the delivery of prodrug-activating enzymes, which are not native to the patient, to the cancer site before the prodrug is administered, ensuring the production of cytotoxic products is local to the cancer site and limiting any off-target effects. Much research has been conducted to determine the optimal way of delivering the prodrug-activating enzymes, covering methods such as antibodies (ADEPT) [5], genes (GDEPT) [6,7], viruses (VDEPT) [8–11] and gold-coated magnetic nanoparticles (MNDEPT) [12,13].

Bacterial nitroreductases (NTRs) have featured prominently in DEPT research because of their ability to readily reduce nitroaromatic prodrugs, such as the widely investigated 5-(aziridin-1-yl)-2,4-dinitrobenzamide (CB1954) prodrug, to form cytotoxic derivatives [6,9,14–19]. Of all the NTRs studied for use in DEPT treatments, the most heavily investigated is the NfnB NTR from *Escherichia coli*, which reduces CB1954 to both its 2- and 4-hydroxlyamine (NHOH) metabolites in a roughly 50:50 ratio [6,20,21]. Of the two products, the 2-NHOH has the greater bystander effect and the 4-NHOH has greater cell killing potency, as it is further reduced by intracellular thioesters to form DNA cross-linking products [11,20–22]. Despite the initial promise of the NfnB/CB1954 combination, the low turnover of the prodrug by NfnB has proven to be a limitation in clinical applications and research has since focussed on identifying new enzyme/prodrug combinations that can be used as alternatives to improve the clinical results for DEPT treatments. This has led to the development of other NTR prodrugs with greater dose potency [23–28] and the identification of other bacterial enzymes that operate in combination with the CB1954 prodrug, such as the YfkO NTR from *Bacillus licheniformis* [14,20,22] and the novel nitroreductases, 1619 and 3024, from *Bacillus cereus* [20].

**Figure 1:**
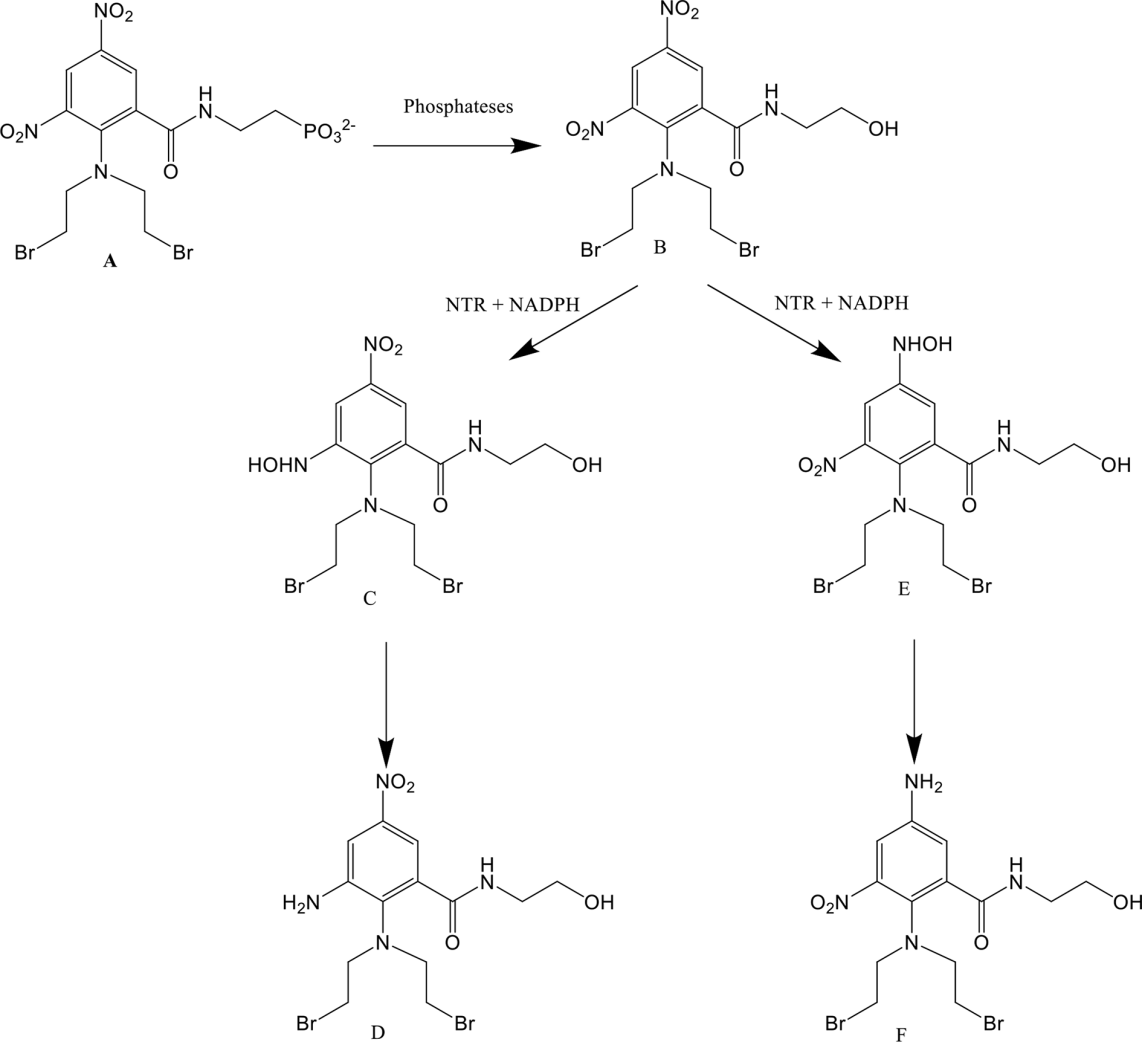
The structure and reactions of the water-soluble pre-prodrug SN28343

Two such alternative prodrugs for use in DEPT treatments include the dinitrobenzamide mustard (DNBM) prodrugs PR-104A (2-[N-(2-bromoethyl)-2-(2-hydroxyethylcarbamoyl)-4,6-dinitroanilino]ethylmethanesulfonate) and its di-bromo analogue SN27686 [23,24,27–30]. When tested, Singleton et al. showed that SN27686 exhibits a much higher dose potency than CB1954 while also displaying a superior bystander effect [25]. Furthermore, when tested in nude mice, the water soluble phosphate pre-prodrug of SN27686; SN28343, achieved a maximum tolerated dose 3.75× higher than the one achieved for CB1954 [25]. The reduction scheme for SN27686 is demonstrated in Figure 1.

SN28343 (**A**), the corresponding alcohol prodrug SN27686 (**B**), the SN27686 2-hydroxylamine derivative formed after the prodrug is reduced by an NTR (**C**) and the subsequent amine product formed after further reduction (**D**), the SN27686 4-hydroxylamine derivative formed after the prodrug is reduced by an NTR (**E**) and the subsequent amine product formed after further reduction (**F**).

The same as SN27686, PR-104A has a water-soluble phosphate pre-prodrug form; PR-104 [23]. The pre-prodrug, PR-104 is rapidly converted to the corresponding DNBM alcohol PR-104A *in vivo* [23]. The phosphate pre-prodrug of PR-104A, PR-104, has advanced through to the clinical trial stage with positive results achieved [6,23,26].

PR-104 (**A**), the corresponding alcohol prodrug PR-104A (**B**), the PR-104A 2-hydroxylamine derivative formed after the prodrug is reduced by an NTR (**C**) and the subsequent amine product formed after further reduction (**D**) the PR-104A 4-hydroxylamine derivative PR-104H formed after the prodrug is reduced by an NTR (**E**) and the subsequent amine product formed after further reduction PR-104M (**F**).

**Figure 2:**
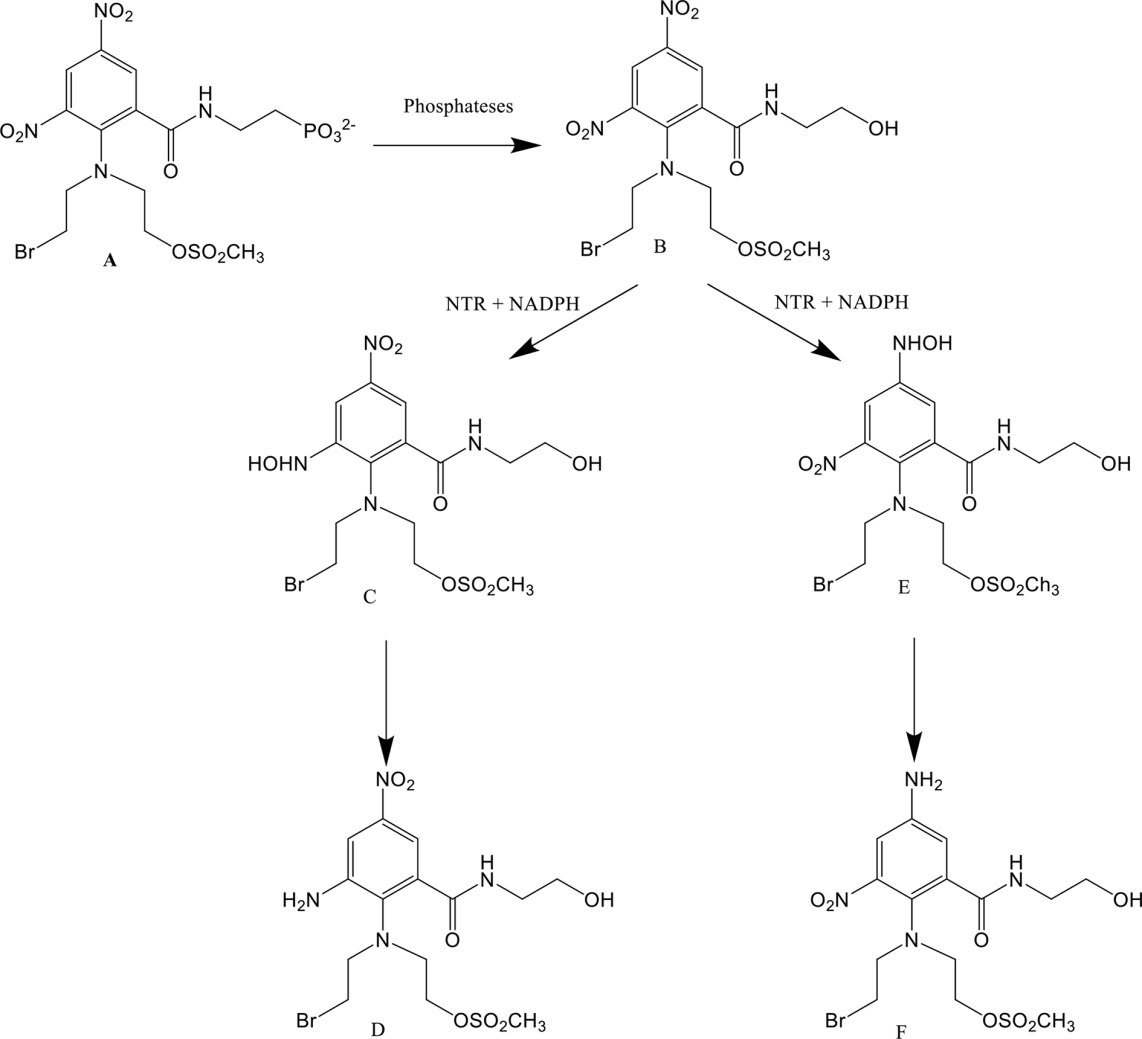
The structure and reactions of the water-soluble pre-prodrug PR-104

The reduction of PR-104 to the cytotoxic product is a two-step process, demonstrated in Figure 2. similar to with SN28343. The alcohol product formed after the first step is so lipophilic that it can penetrate multiple layers of tumour cells; a trait required to reach hypoxic cells [23,24,27]. The reported clinical data for PR-104A shows that that 270 mg/m^2^ can be safely administered to patients on repeated weekly cycles [26], which is much greater than the maximum tolerated dose (MTD) of CB1954, which was shown to be 24 mg/m^2^ by Chung-Faye et al. [8]. The improved MTD for PR-104A, compared with CB1954, is highly promising in terms of improving the results of cancer prodrug therapy treatments as it means patients can safely be treated with over ten times more of this prodrug.

In the present study, it was determined whether the genetically-modified cysteine-tagged NTRs, NfnB-cys and YfkO-cys, were able to effectively reduce the DNBM prodrugs, PR-104A and SN27686, in combination with an NADH cofactor; something which is essential for enzyme/prodrug combinations to be used in novel MNDEPT treatments. While the enzymes and prodrugs have been tested separately in other combinations, this is the first time that they have been tested together for potential use in DEPT strategies. It was also determined whether each of the enzyme/prodrug combinations tested could induce significant cell death in the cancerous cell line, SK-OV-3 without the addition of extracellular cofactor as this would indicate successfully enzyme uptake to access the cells’ intracellular cofactor.

## Methodology

### Chemicals

Unless otherwise stated, chemicals were obtained from Sigma-Aldrich (St. Louis, MO). The prodrugs; PR-104A and its di-bromo analogue SN27686 were synthesised in-house at the Auckland Cancer Society Research Centre (Auckland, New Zealand) [31].

### Transformation

Plasmids of *nfnb-cys* and *yfko-cys* that had been prepared previously [13] were sequence verified by Eurofins Genomics before being transformed into *E.coli* competent cells (Rosetta pLysS (Novagen, Merck, U.K.) and grown on agar plates containing kanamycin (50 µg/ml). The plasmids pET28a+ vector (Novagen, Merck, UK) containing the NTR gene (2 µl) were added to the competent cells (200 µl) and left on ice for 30 min. The samples were then heat shocked at 42°C for 50 s before being placed back on ice for 2 min. The samples were than mixed with sterile Super Optimised Broth media containing glucose (S.O.C. media) (500 µl) (2% tryptone, 0.5% yeast extract, 10 mM NaCl, 2.5 mM KCl, 10 mM MgCl_2_, 10 mM MgSO_4_ and 20 mM glucose) before being incubated at 37°C for 45 min. The sample was then added (125 µl) to sterile agar plates containing kanamycin (50 µg/ml) and spread across the plate using a glass spreader. The plates were then left in a 37°C incubator overnight and checked the following day for colony growth.

### Protein expression

A single colony of bacteria containing the plasmids that had been transformed into *E. coli* Rosetta pLysS (Novagen, Merck, U.K.) was picked and grown in 5 ml of Luria-Bertani (LB) broth with kanamycin (50 µg/ml) overnight at 37°C, this was then transferred into 500 ml of sterilised LB broth with kanamycin (50 µg/ml) and grown to an OD of 0.6 at 37°C whilst shaking at 180 rpm. After which, isopropyl β-D-1-thiogalactopyranoside (IPTG) (2 ml, 100 mM) was added and the cultures were left to grow for another 4 h. Next, the cultures were centrifuged for 10 min at 8,000 rpm (5400 × ***g***) and 4°C to give pellets containing the expressed proteins.

### Protein purification

The protein pellets were resuspended in binding buffer (10 ml, potassium phosphate 50 mM, NaCl 400 mM, Imidazole 10 mM) and sonicated before being centrifuged at 20,000 rpm (1,300 ×*** g***) to pellet any cell debris. The yellow supernatant was then purified using metal ion affinity chromatography (Ni2+) (HiTrap chelating column, Amersham Biosciences, U.K.) and eluted with an imidazole gradient, all the fractions were collected in 5 × 1 ml aliquots and kept for analysis using SDS-PAGE. Due to the denaturing nature of SDS-PAGE, proteins migrated through the gel as monomers (approximately 28 kDa for NfnB-cys and 30 kDa for YfkO-cys). Next, the IMAC fractions containing the proteins of interest were incubated for an hour with Flavin Mononucleotide (FMN) (5.6 mM, 1 ml) before being purified from imidazole into phosphate buffer (50 mM, pH 7.4) using a PD-10 desalting column (Amersham Biosciences, U.K.). The concentration of protein was determined using the Bradford method. The Bradford assay was carried out using Quick Start Bradford Dye Reagent from Bio-Rad, U.K. with Bovine Serum Albumin (BSA) being used as the standard for calibration, following the manufacturer’s instructions. Protein yield was generally 2–5 mg/ml pure protein [32].

### Enzyme reactivity with DNBM prodrugs

The ability of the purified proteins to reduce the DNBM prodrugs was confirmed following the method previously described by V. Gwenin et al. [13] with minor modifications. Briefly, the proteins were incubated with NADH (300 μM) and the prodrug (100 μM) in phosphate buffer (PB) (50 mM, pH 7.2) and scanned using UV-visible spectroscopy every 90 s for 15 min. For active NTR/prodrug combinations, prodrug consumption was measured at 400 nm [13,20].

### Prodrug kinetics studies

All the kinetics experiments were run using a Thermo Scientific Varioscan 96-well plate microplate reader. To determine the Michaelis–Menten kinetic parameters of PR-104A and SN27686 when using NfnB-cys or YfkO-cys, prodrug consumption at 400 nm was measured over time. In each well of the 96-well plate, prodrug (0.1–20 mM), NADH (4 mM) and PB (50 mM, pH 7.2) were combined and incubated at 37°C for 3 min before the purified NTR (20 μg/ml) was added. The dimethyl sulfoxide (DMSO) solvent concentration was always kept constant at 5% v/v to avoid any negative effect [14].

The amount of the prodrug consumed per second was calculated in Microsoft Excel using the change of absorbance over 20 s and the molar extinction coefficient of the prodrug (ε = 5600 M^−1^cm^−1^ at 400 nm for PR-104A and ε = 6400 M^−1^ cm^−1^ at 400 nm for SN27686). The data were then transferred to SigmaPlot 12 (SPSS, Systat Software Inc.) where a non-linear regression tool was used to generate a Michaelis–Menten hyperbolic curve and a report containing the important kinetic information of the system under test.

### HPLC

All experiments were conducted on a HPLC machine (Dionex Ultimate 3000 HPLC system, ThermoScientific, U.S.A.) using a C18 column for analysis (Waters Spherisorb® 5 µm ODS2 4.6 mm × 250 mm C18 column, U.K.). The instrument was run using the following parameters; 50 µl injection volume, a fixed column oven and sampler temperate of 25°C, a run length of 45 min and the UV wavelength for detection was 254 nm [21,24]

HPLC samples were prepared in a 15 ml falcon tube covered in foil: NADH (60 μl, 20 mM), prodrug (10 μl, 100 mM), NTR (116 μg/ml) and made up to 1080 μl with PB (50 mM, pH 7.2). The reaction mixture was incubated at room temperature for 15 min before being de-gassed with nitrogen for 15 min. Next 700 μl of the de-gassed mixture was placed in a chromacol select 2 ml vial and placed in the HPLC machine. The solvent consisted of an acetonitrile/water mixture, beginning with 10% acetonitrile and increasing by 1% acetonitrile per minute. After 20 min, this gradient increase to 40% acetonitrile per minute, reaching 100% after 22 min. Eluents were scanned at 254 nm and product peaks were identified by comparisons with all reagents run individually as standards as well as a negative control containing all reagents with the exception of the prodrug.

HPLC experiments done to assess the product ratio were done using a single injection onto the HPLC machine after 30 min reaction time split between a 15-min incubation and a 15-min degas. The time-dependent HPLC experiments were done in the same way with a 15-min incubation and a 15-min degas of the reaction mixture prior to injection onto the HPLC machine with further injections of the same reaction been carried out every 45 min from that point onwards.

### Cell viability assays

The MTT assay was performed following the method of Mosmann, 1983 [33] with slight modification. Briefly, SK-OV-3 (ECACC 91091004) cells (Sigma-Aldrich, U.K.) were seeded in 96-well plates (Corning, U.S.A.) at a density of 1000 cells per well, in 100 µl Dulbecco’s Modified Eagle Medium (DMEM) containing 10% FBS and 1% penicillin/streptomycin and were left to attach overnight in a 5% CO2 incubator at 37°C. After 16 h, medium was carefully aspirated, and medium (50 µl) containing prodrug (20 mM) was added. Next, medium (50 µl) containing a set amount of purified enzyme was added and after 4 h, the medium was removed, and cells were replenished with complete DMEM (100 µl). After 48 h, MTT (20 µl 5 mg/ml) was added to each well and incubated at 37°C for 4 h under 5% CO_2_. The purple formazan crystals formed were dissolved in 100 µl of DMSO after removing the media carefully and the absorbance was read at 570 nm in a Thermo Scientific Varioscan 96-well plate microplate reader.

## Results

### Protein expression and purification

The pET28a+ vector contains a his-tag which is inserted into all the recombinant proteins for ease of purification using metal ion affinity chromatography (IMAC), with further genetic modifications performed to insert a cysteine-tag made up of 6 N-terminal cysteine residues to facilitate immobilisation onto gold-coatedSupplement nanoparticles in MNDEPT treatments [12,13,34]. Both the NfnB-cys and YfkO-cys proteins were successfully purified and obtained at a yield of up to 10 mg/ml. Each of the purified NTRs were confirmed to be active with both prodrugs following the same method as was described previously by Gwenin et al. [13,20] utilizing UV/Visible spectroscopy to monitor the enzymatic reduction of the prodrug over time.

### Enzymatic reduction of the DNBM prodrugs

Initially, enzyme reactivity to PR-104A and SN27686 in the presence of NADH was confirmed following the method previously described by Gwenin et al. and Ball et al. with one notable difference [13,20,35]. In previous work using the CB1954 prodrug, product formation could be observed using UV/Vis spectroscopy at 420 nm. In the case of the DNBM prodrugs being used here, prodrug consumption is observed at 400 nm using UV/Vis spectroscopy. NADH consumption can be seen (provided in Supplementary Figure S1) at 340 nm and Prodrug consumption can be observed at 400 nm illustrating an active enzyme/prodrug combination. All four enzyme/prodrug combinations tested in this study were proven to be active in this way. (The activity scans for NfnB-cys with SN27686, YfkO-cys with SN27686 and Pr-104A are provided in Supplementary Figures S2, S3, and S4, respectively). As neither PR-104A nor SN27686 have been tested previously with the genetically modified cys-tagged NTRs, proving them to be active combinations in this way is a result of vital importance as it is the first time that these have shown to be active combinations. Next, the Michaelis–Menten kinetic parameters were determined for both PR-104A and SN27686 in combination with the NfnB-cys NTR (Table 1).

**Table 1:** Michaelis–Menten kinetic data obtained for NfnB-cys and YfkO-cys by varying concentrations of PR-104A or SN27686 prodrug

Enzyme	Prodrug	*V*_max_ μM s^−1^	*K*_m_ μM	*k*_cat_ s^−1^	*k*_cat_/*K*_m_ μM ^-1^ s^−1^
NfnB-cys	PR-104A	12.8 ± 1.5	3100 ± 400	35.0 ± 0.48	0.011 ± 3.6 × 10^−3^
YfkO-cys	PR-104A	11.4 ± 1.6	1900 ± 350	34.2 ± 0.25	0.018 ± 4.6 × 10^−3^
NfnB-cys	SN27686	0.7 ± 0.2	250 ± 80	2.0 ± 0.35	0.008 ± 3.1 × 10^−3^
YfkO-cys	SN27686	1.7 ± 0.6	420 ± 120	5.2 ± 0.16	0.012 ± 2.8 × 10^−3^

Prodrug consumption was measured at at 400 nm. All the Michaelis–Menten kinetics data presented below are obtained using the averages of at least five repeats. Here, *V*_max_ represents the maximum rate of the system, *K*_m_ is the enzyme concentration at half of *V*_max_ and* k*_cat_ is the enzyme turnover rate.

The *k*cat/*K*m term is the Michaelis–Menten specificity constant and can be used to evaluate the efficiency of each enzyme/prodrug combination (where *k*cat is the enzyme turnover number and *K*m is the substrate concentration when the system is at half its total rate), with higher values of *k*cat/*K*m indicating a more efficient combination [11,36]. The kinetic data obtained here was a good match for that seen in the literature with Prosser et al. reporting a *k*cat/*K*m value of 0.013 μM s^−1^ for the NfnB/PR-104A combination [21] compared with a value of 0.011 μM s^−1^ obtained here for the NfnB-cys/PR-104A combination. The fact that the YfkO-cys/PR-104A combination yielded a higher result for *k*cat/*K*m than the NfnB-cys/PR-104A combination (0.018 μM s^−1^ compared with 0.011 μM s^−1^) whilst also having a lower value for *K*m (1935 μM compared with 3172 μM) is a promising result, in that this combination is more efficient than the NfnB-cys/PR-104A combination and is more effective at lower prodrug concentrations which is a result of clinical importance. Furthermore, upon analysis of the kinetic data obtained for the SN27686 it is clear that this prodrug is more efficient in its reaction with YfkO-cys than with NfnB-cys (*k*cat/*K*m of 0.012 μM s^−1^ for the YfkO-cys/SN27686 combination compared with 0.008 μM s^−1^ for the NfnB-cys/SN27686 combination). However, in the case of SN27686, it was the NfnB-cys NTR that demonstrated a higher affinity for the prodrug with a lower *K*m value (257 μM for the NfnB-cys/SN27686 combination compared to 420 μM for the YfkO-cys/SN27686 combination). These results further emphasize the potential of YfkO-cys for use in DEPT strategies as it shows a higher efficiency than the NfnB-cys NTR with both DNBM prodrugs tested.

### HPLC analysis

Because NfnB-cys and YfkO-cys have not previously been tested with the DNBM prodrugs, PR-104A and SN27686, HPLC was performed to try and identify the products formed in each reaction. As per the literature the chromatograms were analysed at 254 nm [21] and the peaks were identified by comparisons with all reagents run individually as standards, as well as a prodrug-negative controls which contained all reagents with the exception of the prodrug in question.

This enzymatic conversion forms multiple products which absorb at 254 nm. Here NADH was the cofactor.

**Figure 3:**
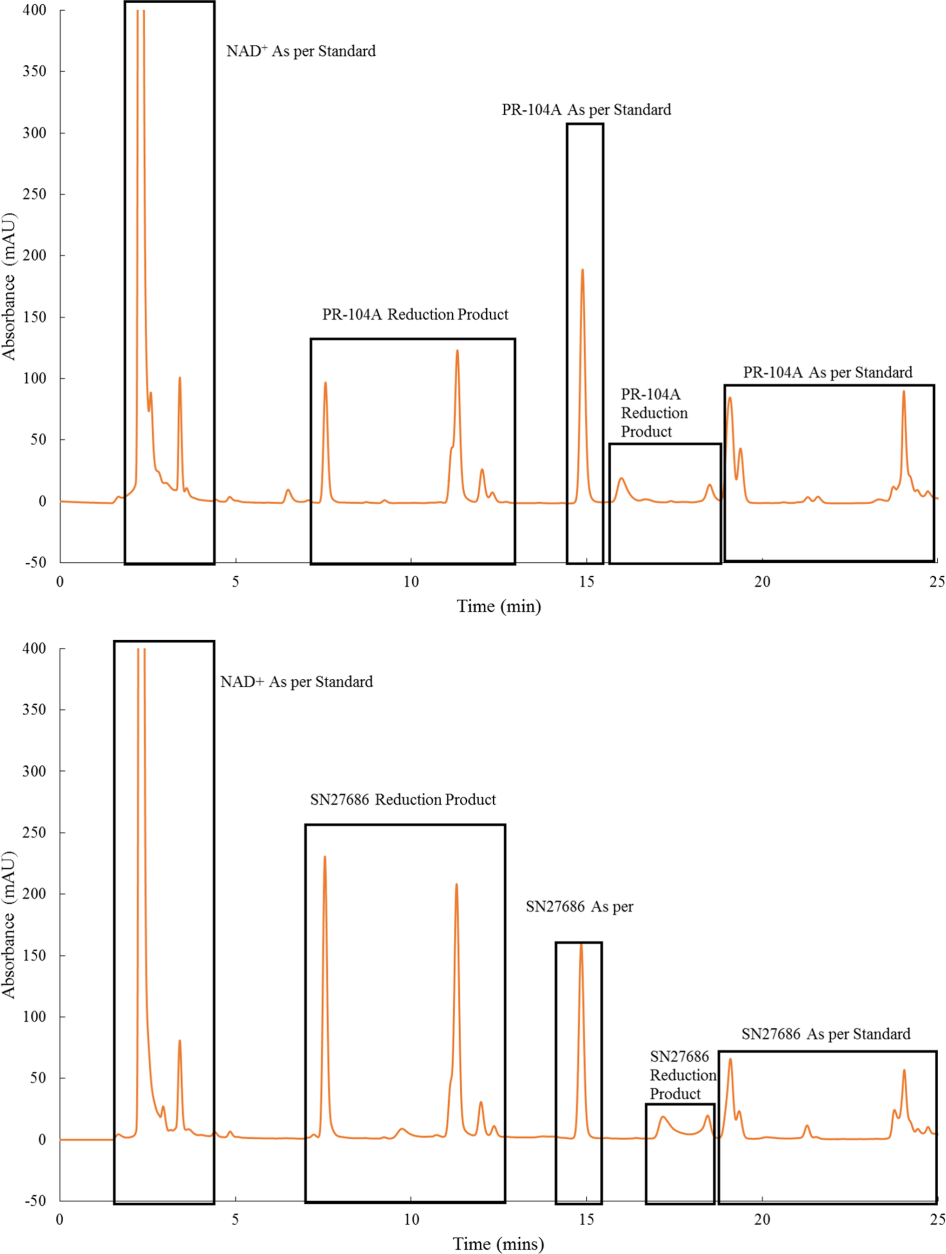
HPLC chromatogram confirming the enzymatic conversion of (top) PR-104A by NfnB-cys and (bottom) SN27686 by NfnB-cys

The chromatogram (Figure 3, top) illustrates that NfnB-cys can effectively reduce the PR-104A prodrug with reduction products seen to elute from 7.5 to 12 min and from 17 to 18 min. The additional peaks that are seen to elute from 2 to 4 min have been shown to be the NAD+ caused by the oxidation of the NADH cofactor in the enzymatic reaction. The reduction products that appear from 7.5 to 12.5 min appeared to be the intermediate reduction products such as the para-hydroxylamine product, PR-104H [23], Also, when monitored over time the absorbance of the peaks decreased, which seemed to indicate that these products were being further reduced to other products as the reaction was allowed to progress. When tested in the same way using HPLC, the YfkO-cys/PR-104A combination produced the same peak pattern on the chromatogram as were observed for the NfnB-cys/PR-104A combination. The produced chromatograms were compared against reference standard chromatograms of nitro reductase standard, NADH standard, PR-104A standard and DMSO standard which are provided all overlayed in Supplementary Figure S4.

The HPLC confirmed that the PR-104A prodrug was being enzymatically reduced to what appeared to be the para-hydroxylamine and -amine derivatives. However, without access to other analytical tools such as mass spectrometry the exact identity of the products cannot be verified. Next, the enzymatic reactions of the cys-tagged NTRs with the SN27686 prodrug were assessed by HPLC. This enzymatic conversion forms multiple products which absorb at 254 nm. Here NADH was the cofactor.

The elution patterns for the enzymatic reactions using the SN27686 prodrug (Figure 3, bottom) were extremely similar to those seen previously for PR-104A with multiple peaks relating to the reduction products eluting from 8 to 13 minutes and 16 to 18 minutes respectively. As was the case in the HPLC results obtained when testing PR-104A, SN27686 can be seen to be being reduced by both enzymes, NfnB-cys and YfkO-cys, however the exact identity of the reduction products cannot be confirmed without access to additional analytical tools. The characterization of these reduction products could form the basis of a future study as confirming the products present will be an important step in advancing DEPT treatments using the DNBM prodrugs.

### Cell viability assays

**Figure 4:**
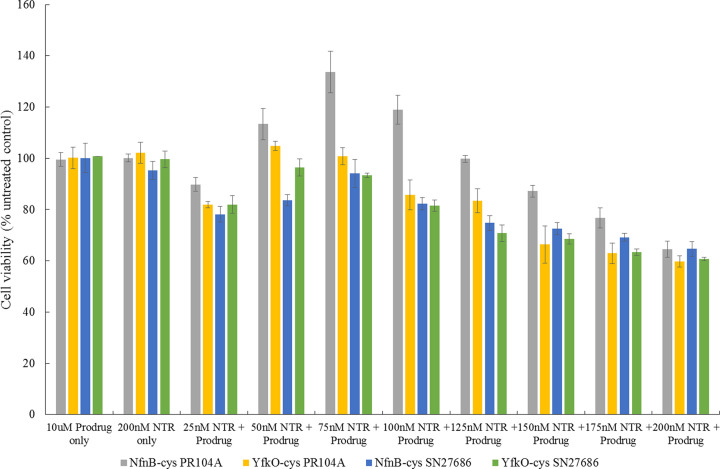
Percentage cell survival relative to untreated control cells of SK-OV-3 cells

Percentage cell viability of SK-OV-3 cells, relative to untreated controls, was determined in the presence of increasing concentrations of enzyme, NfnB-cys or YfkO-cys, in combination with a fixed concentration (10 µM) of prodrug, either PR-104A or SN27686 (Figure 4). Controls were performed using cell culture medium (DMEM) only, enzyme only (NfnB-cys or YfkO-cys) and prodrug only (PR-104A or SN27686). The data points were plotted based on the averages taken from at least three repeats with error bars representing the standard deviation. The prodrug concentration was fixed at 10 µM to allow the results to be directly compared with work we have done using the CB1954 prodrug. This concentration was chosen previously as the concentration of CB1954 used *in vivo* cannot exceed 10 µM based on the maximum tolerated dose observed in clinical trials using the drug [6,8,9,19,37].

Assay conditions consisted of a 4-h incubation with either: prodrug only, enzyme only and increasing concentrations of either NfnB-cys or YfkO-cys (25–200 nM) in presence of a fixed concentration of prodrug, PR-104A or SN27686, (10 µM). Data points were analysed for their statistical significance using *F*-test with a *P*-value of (>0.05), error bars are ±1 standard deviation, and (*n*) is the number of repeats each data point had.

It is of promise that no significant cell kill was observed when the cells were treated with the controls of either of the prodrugs alone or either of the enzymes alone. All enzyme/prodrug combinations showed an immediate response to treatments, with a significant decrease in the cell viability percentage of about 15–20% upon the addition of 25 nM of enzyme in combination with one of the DNBM prodrugs. As there was no extracellular NADH cofactor added in this experiment this result is particularly promising as it indicates that the enzymes were able to be uptaken into the SK-OV-3 cells and access the intracellular cofactor available within them to facilitate their reactions with the DNBM prodrugs. It is evident that there occurred an initial decrease in the cell killing potency of all four treatment combinations tested as the enzyme concentration was increased from 25 nM before the cell killing potency began to increase again as the enzyme concentration approached 200 nM and whilst this may initially appear to be contradictory it is a phenomenon which is well documented in the literature and is known as the Hormetic effect [38]. This is evident in the biphasic response of the SK-OV-3 cells to increasing concentrations of enzyme used in the cell viability assay, leading to a characteristic inverted U-shaped response on the graph [35]. This effect is visibly much more prominent in the treatments using the PR-104A prodrug compared to those using SN27686. It is evident that the treatments using the SN27686 prodrug always performed on par with or superior to treatments using PR-104A in terms of the cell killing potency. This result could hold some significance when selecting which enzyme/prodrug combinations should be tested further for their MNDEPT therapeutic potential.

## Discussion

In this report, the genetically modified cysteine-tagged enzymes, NfnB-cys and YfkO-cys, were tested in combination with two promising DNBM prodrugs, PR-104A and SN27686, for their potential use in novel MNDEPT cancer chemotherapy treatments.

Firstly, it had to be ascertained whether the cys-tagged NTRs, NfnB-cys and YfkO-cys, are able to reduce the prodrugs, PR-104A and SN27686, in combination with an NAD(P)H cofactor as each of these enzyme/prodrug combinations have never before been tested in the literature. It was shown that both prodrugs could be effectively reduced by both NfnB-cys and YfkO-cys when using NADH as the cofactor, a result of clinical significance as NADH is more abundant intracellularly than NADPH [35,39]. Next, the Michaelis–Menten kinetic parameters for each enzyme/prodrug combination were determined in the presence of an NADH cofactor (Table 1). It was evident from the kinetics data presented that the YfkO-cys enzyme in particular held great promise for use in MNDEPT treatments in combination with the DNBM prodrugs as it demonstrated a higher efficiency (higher *K*cat/*K*m) than NfnB-cys in its reactions with both prodrugs and in the case of PR-104A, the YfkO-cys enzyme also demonstrated a far greater affinity (lower* K*m) for the prodrug than was seen when using NfnB-cys.

HPLC was used to analyse the products formed after each of the prodrugs had been reduced by the NTRs using an NADH cofactor. It was clear from the chromatograms presented that both the NfnB-cys and YfkO-cys NTRs were able to reduce PR-104A (Figure 3, top) and SN27686 (Figure 3, bottom) forming a series of reduction products for each prodrug. Prosser et al. reported that, when assessing the metabolites produced via reduction of PR-104A by a range of NTRs that, all but one NTR tested was found to reduce PR-104A exclusively at the NO_2_ group para to the mustard moiety [21]. Patterson et al. have reported that the NO_2_ group ortho to the mustard group can be reduced under hypoxic conditions leading to the formation of a non-toxic tetrahydro-quinoxaline derivative [23]. However, because the HPLC traces show the formation of several uncharacterized products it is difficult to draw definitive conclusions as to the nature of the products detected in the HPLCs in the present study without access to other analytical techniques such as mass spectrometry and this could form the basis of a future study.

As well as proving that both genetically modified NTRs were able to reduce both DNBM prodrugs it was important to demonstrate that each of the enzyme/prodrug combinations could induce a significant cell death in a cancer cell line, in this case the ovarian cancer cell line SK-OV-3. It was decided that the cell viability experiments would be conducted without the addition of extracellular cofactor so that if a significant cell killing was observed it could be attributed to the successful uptake of the enzymes into the cancer cells thus accessing the intracellular cofactor available there. While this might lead to a lower observed cell kill than other experiments seen in the literature, it means that the cell killing potential of the combinations presented here isn’t artificially inflated by the addition of extracellular cofactor. When comparing the two enzymes in terms of their cell killing potency (Figure 4) when combined with either prodrug it is difficult to distinguish between the two as typically the cell viability percentages reported are within error of each other making it reasonable to suggest that both enzymes are equally able to induce cell death in SK-OV-3 cells in a 2D cell treatment model.

In conclusion, two promising DNBM prodrugs, PR-104A and SN27686, have been identified as potential candidates for use in future MNDEPT cancer chemotherapy treatments. Both prodrugs were shown, for the first time, to be able to be reduced by the genetically modified NTRs, NfnB-cys and YfkO-cys, that have been developed within the ARCH research group. In terms of the Michaelis–Menten kinetics results that were produced for each enzyme/prodrug combination; the YfkO-cys/PR-104A combination was highlighted as being a combination which showed great promise.

## Future direction

As both of the DNBM prodrugs have been reported in the literature to produce hypoxic specific toxicity, future work could extend into testing each of the enzyme/prodrug combinations presented in this study in a 3D cell culture model so that this hypoxic specific toxicity can be represented in the dataset as this was not possible within this study. It is however evident from these data that both enzymes were able to successfully be uptaken into the SK-OV-3 cells to access the intracellular NADH cofactor and facilitate the reactions with the DNBM prodrugs.

When it came to the cell culture experiments conducted in this study, all combinations displayed the Hormetic effect with an initial cell kill observed which decreased as the enzyme concentration increased but then after a certain point began to increase again. SK-OV-3 treatments performed using the SN27686 prodrug seemed to show a marginally higher level of cell kill compared to those done with PR-104A and it would be of great interest to see if this trend remains, or even increases, when each of the combinations are tested using 3D cell culture models in the future. Future work would also include determining the *in vivo* fate of the prodrugs.

## Informed Consent Statement

Any research article describing a study involving humans should contain this statement. Please add “Informed consent was obtained from all subjects involved in the study.” OR “Patient consent was waived due to REASON (please provide a detailed justification).” OR “Not applicable.” for studies not involving humans. You might also choose to exclude this statement if the study did not involve humans. Not applicable.

## Supplementary Material

Supplementary Figures S1-S5

## Data Availability

All supporting data are included within the main article and its supplementary files.

## Abbreviations

BSA: Bovine Serum Albumin
DEPT: directed enzyme prodrug therapy
DNBM: dinitrobenzamide mustard
FMN: Flavin Mononucleotide
IPTG: isopropyl β-D-1-thiogalactopyranoside
MTD: maximum tolerated dose
NTR: nitroreductase
