# Supplementary material for: The dinitrobenzamide mustard prodrugs, PR-104A and SN27686, for use in a novel MNDEPT cancer prodrug therapy approach
Source: Biosci Rep. 2023 Apr 25;43(4):BSR20230627. doi: 10.1042/BSR20230627 (PMC10126811; doi:10.1042/BSR20230627)
Supplement: Supplementary Figures S1-S5 [file BSR-2023-0627_supp.pdf]

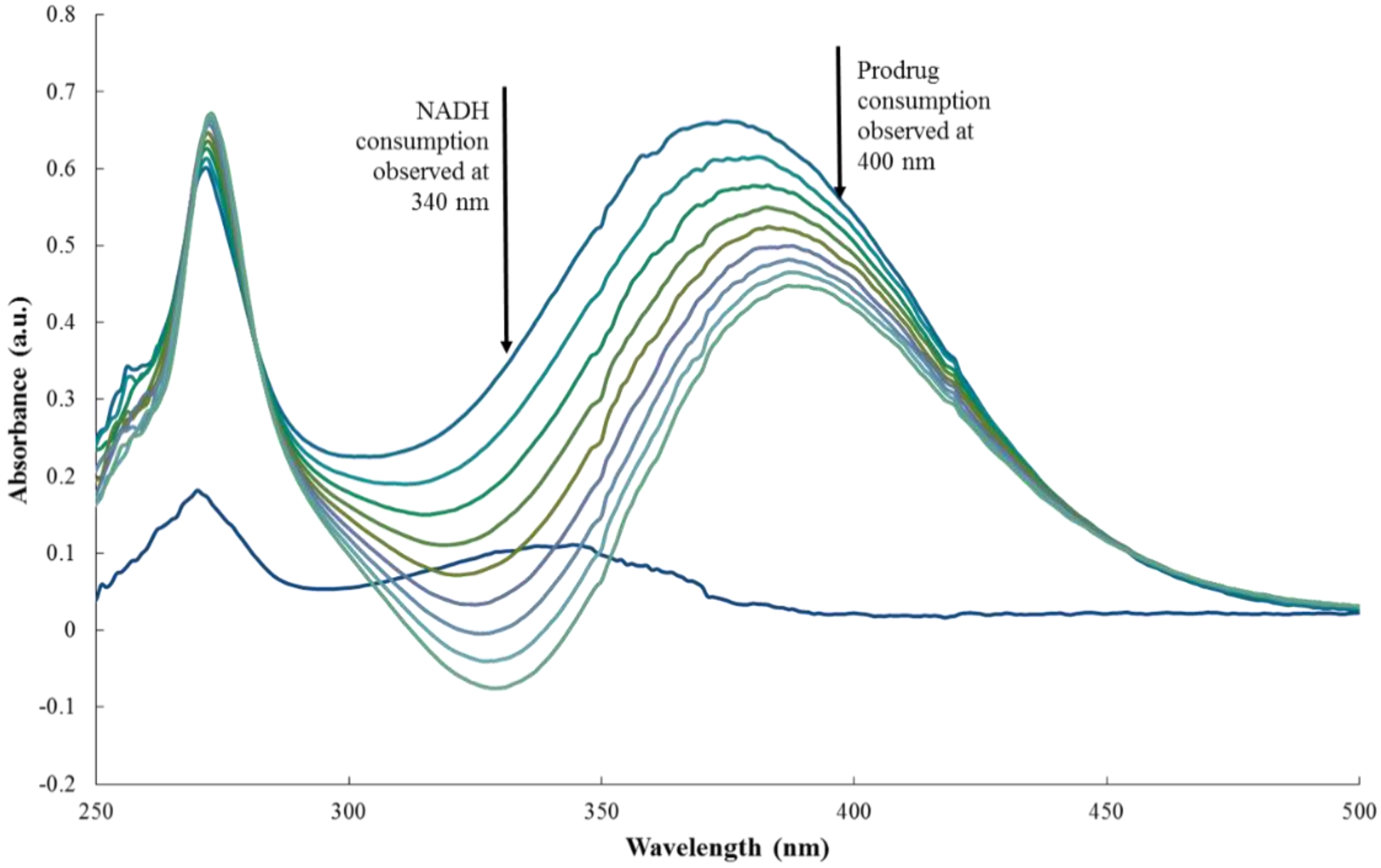

Supplementary figure 1. UV/Vis spectra showing the consumption of PR-104A and NADH in the presence of the NfnB-cys nitroreductase over time

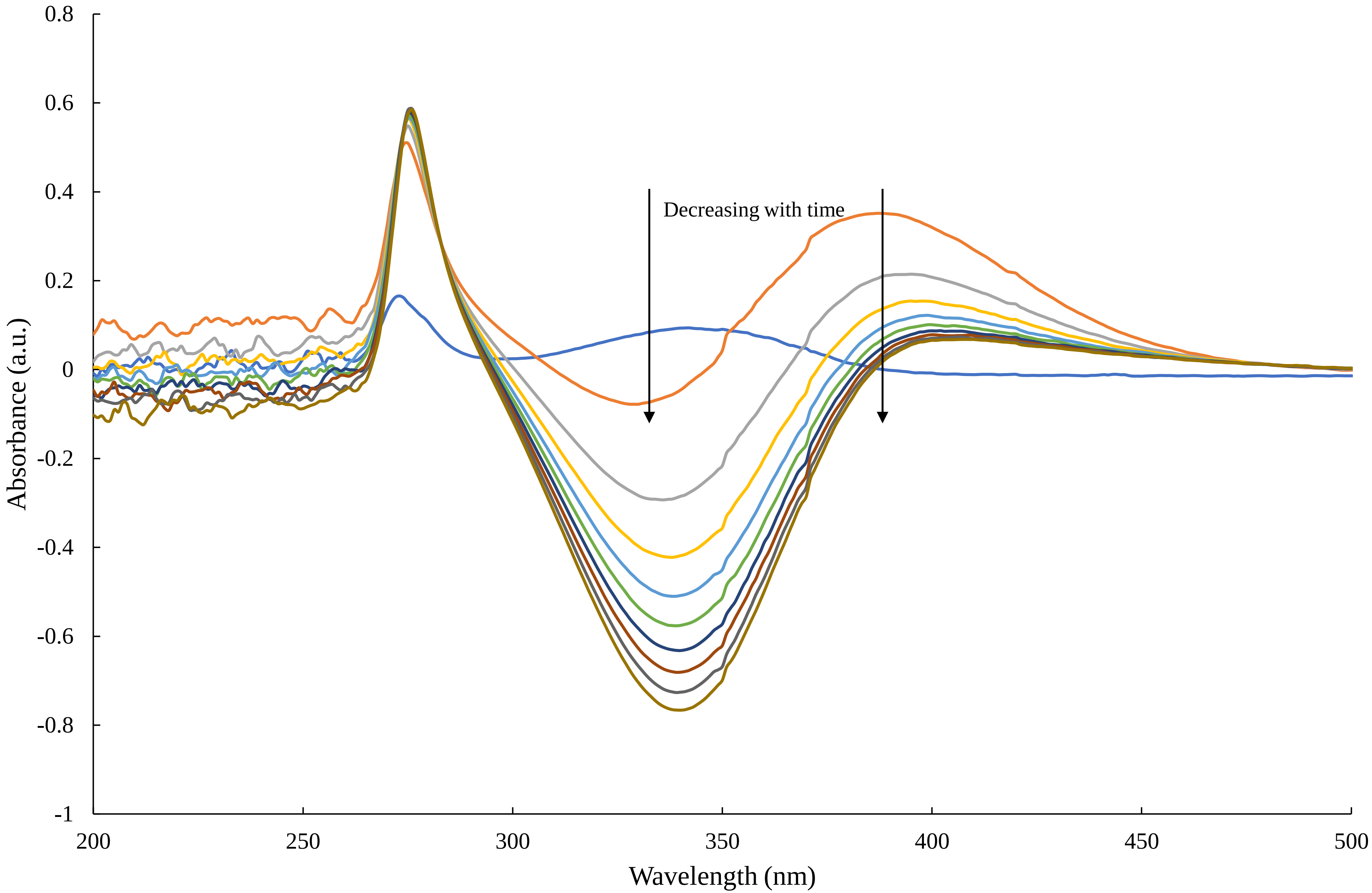

Supplementary figure 2. **UV/Vis spectra showing the consumption of SN27686 and NADH in the presence of the NfnB-cys nitroreductase over time.**

NADH consumption can be seen at 340 nm and Prodrug consumption can be observed at 400 nm.

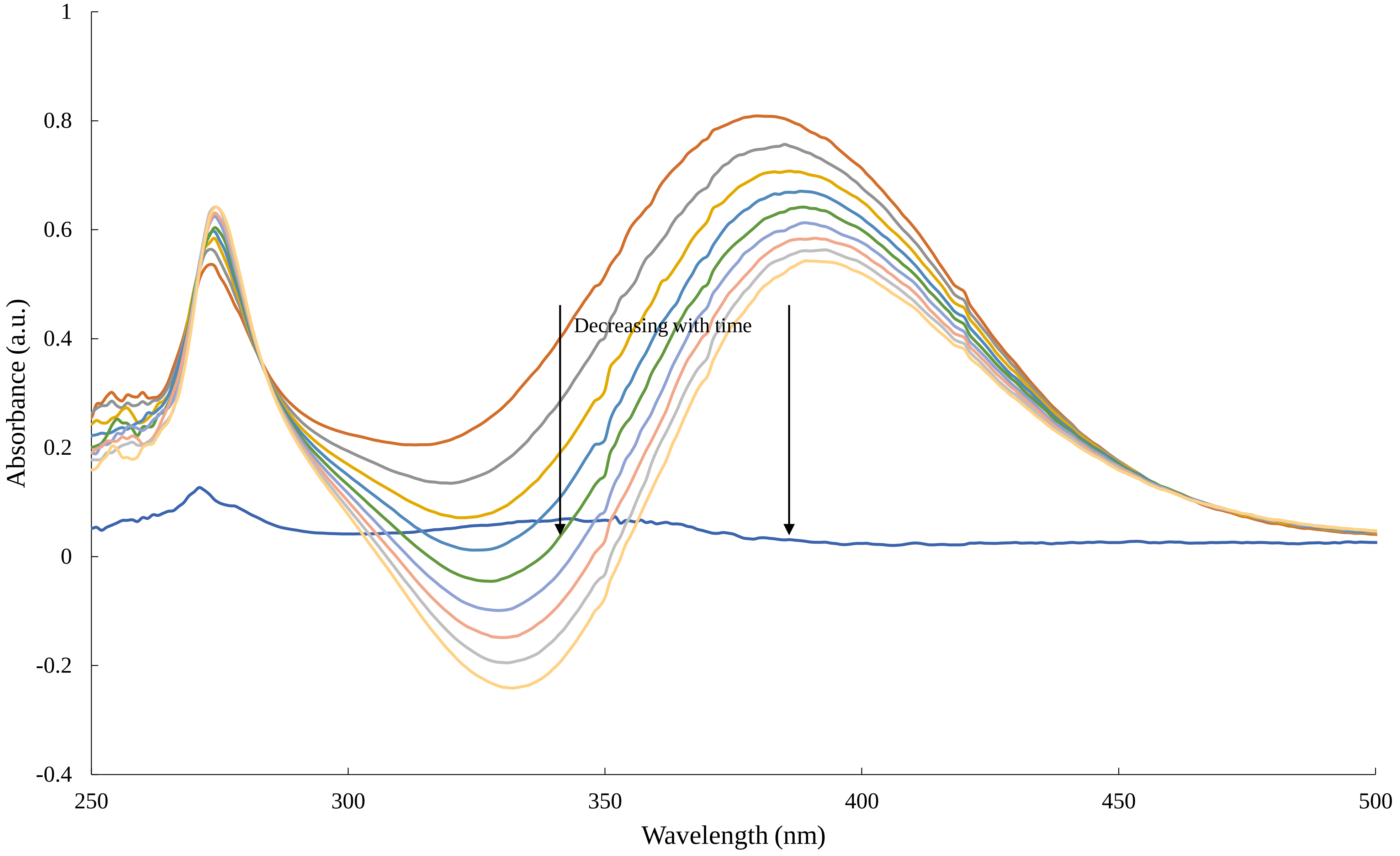

Supplementary figure 3. **UV/Vis spectra showing the consumption of SN27686 and NADH in the presence of the YfkO-cys nitroreductase over time.** NADH consumption can be seen at 340 nm and Prodrug consumption can be observed at 400 nm.

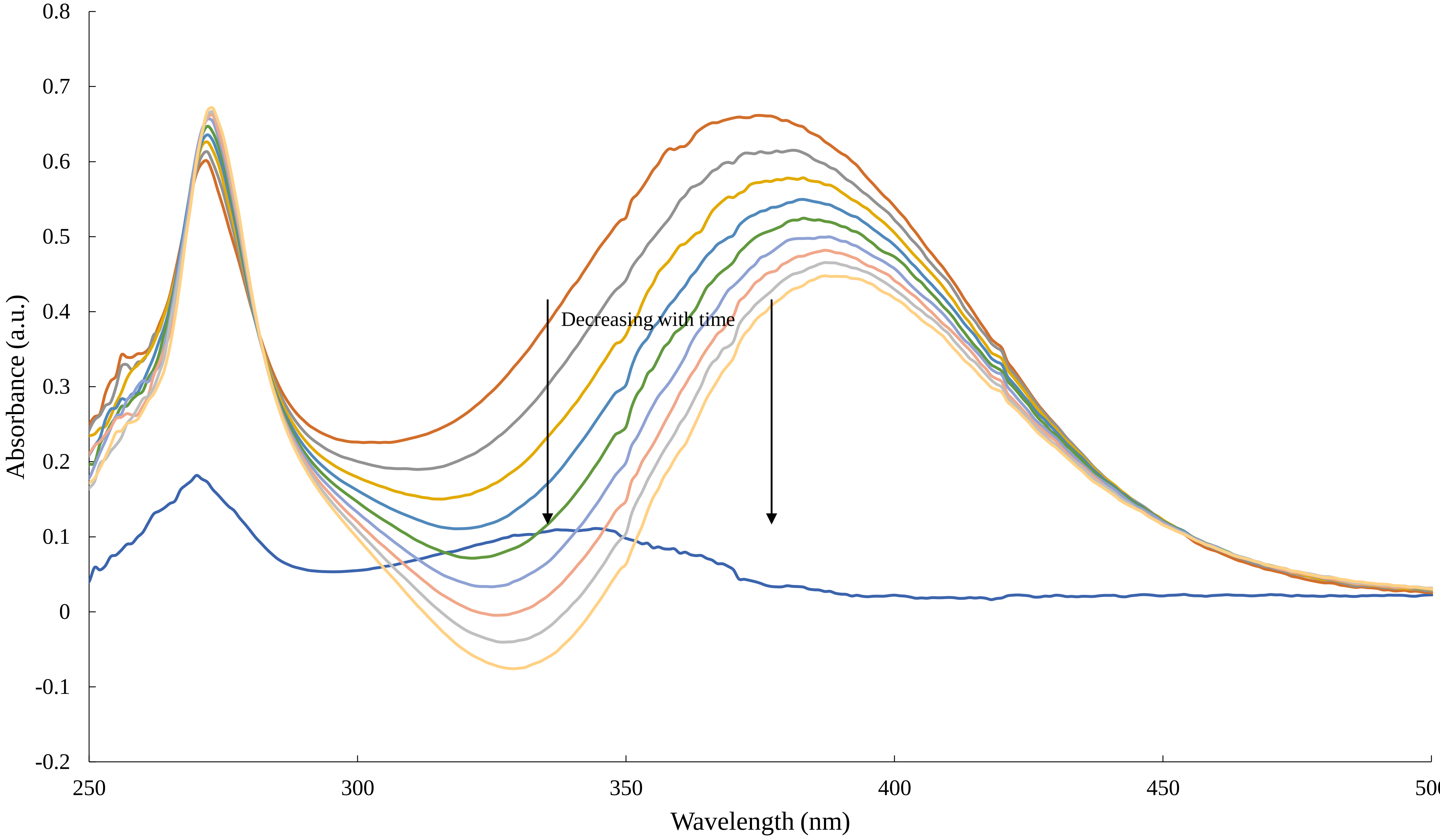

Supplementary figure 4. **UV/Vis spectra showing the consumption of PR-104A and NADH in the presence of the YfkO-cys nitroreductase over time.** NADH consumption can be seen at 340 nm and Prodrug consumption can be observed at 400 nm.

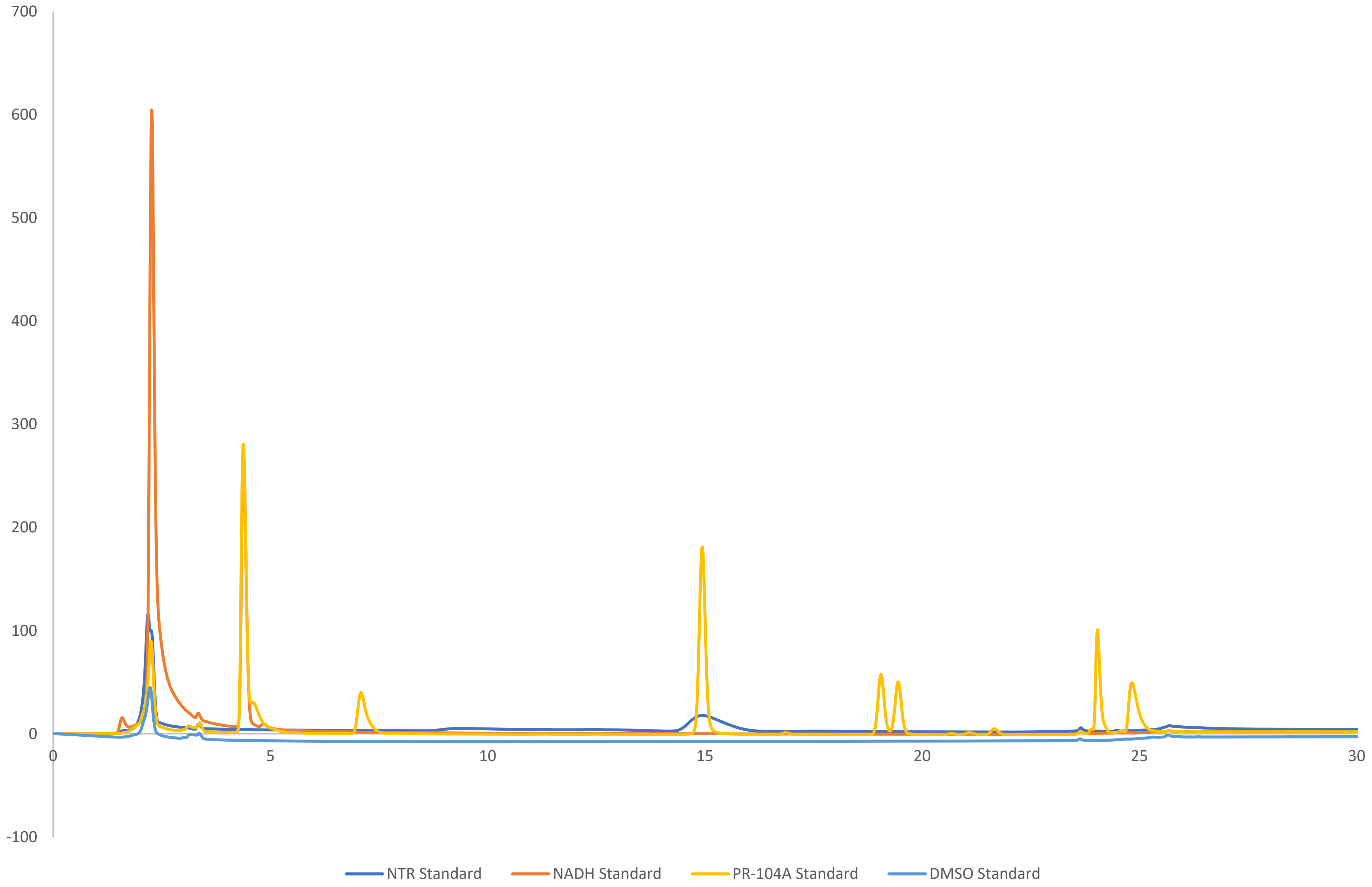

Supplementary figure 5. HPLC chromatogram standards
